# Synthesis and Study of Antifungal Properties of New Cationic Beta-Glucan Derivatives

**DOI:** 10.3390/ph14090838

**Published:** 2021-08-24

**Authors:** Kamil Kaminski, Magdalena Skora, Paweł Krzyściak, Sylwia Stączek, Agnieszka Zdybicka-Barabas, Małgorzata Cytryńska

**Affiliations:** 1 Faculty of Chemistry, Jagiellonian University, Gronostajowa 2 St., 30-387 Krakow, Poland; 2 Department of Infections Control and Mycology, Chair of Microbiology, Jagiellonian University Medical College, Czysta 18 St., 31-121 Krakow, Poland; magdalena.skora@uj.edu.pl (M.S.); pawel.krzysciak@uj.edu.pl (P.K.); 3 Department of Immunobiology, Institute of Biological Sciences, Faculty of Biology and Biotechnology, Maria Curie-Sklodowska University, Akademicka 19 St., 20-033 Lublin, Poland; s.staczek@poczta.umcs.lublin.pl (S.S.); barabas@poczta.umcs.lublin.pl (A.Z.-B.); cytryna@poczta.umcs.lublin.pl (M.C.)

**Keywords:** antifungal, beta-glucan, polycations, *Galleria mellonella* model

## Abstract

The interaction of positively charged polymers (polycations) with a biological membrane is considered to be the cause of the frequently observed toxicity of these macromolecules. If it is possible to obtain polymers with a predominantly negative effect on bacterial and fungal cells, such systems would have great potential in the treatment of infectious diseases, especially now when reports indicate the growing risk of fungal co-infections in COVID-19 patients. We describe in this article cationic derivatives of natural beta-glucan polymers obtained by reacting the polysaccharide isolated from *Saccharomyces boulardii* (SB) and *Cetraria islandica* (CI) with glycidyl trimethyl ammonium chloride (GTMAC). Two synthesis strategies were applied to optimize the product yield. Fungal diseases particularly affect low-income countries, hence the emphasis on the simplicity of the synthesis of such drugs so they can be produced without outside help. The three structures obtained showed selective anti-mycotic properties (against, i.e., *Scopulariopsis brevicaulis*, *Aspergillus brasiliensis*, and *Fusarium solani*), and their toxicity established using fibroblast 3T3-L1 cell line was negligible in a wide range of concentrations. For one of the polymers (SB derivative), using in vivo model of *Aspergillus brasiliensis* infection in *Galleria mellonella* insect model, we confirmed the promising results obtained in the preliminary study.

## 1. Introduction

Fungal infections are one of the most common diseases affecting people all over the world. Fungi are responsible for over a billion infections globally and cause more than 1.5 million deaths worldwide [1]. Most people during their lifetime will suffer from superficial or mucocutaneous fungal infections, which generally do not have serious health consequences but often significantly reduce the quality of life. However, millions will develop life-threatening deep infections (invasive fungal infections—IFI), frequently a multiorgan mycosis including fungaemia, that are much more difficult to diagnose and treat. Mortality rates of IFI depend on the causative agent and the presence of host predisposing factors but often exceed 50% [2]. An increase in the frequency of mycoses has been observed for years. Paradoxically it is related to the progress of medicine, which allows patients with serious underlying diseases to be kept alive, contributing directly or as a result of therapy to increased susceptibility to mycoses (e.g., HIV infection and AIDS, iatrogenic immunosuppression, invasive medical procedures, antibiotic therapy, steroid therapy, *diabetes mellitus*).

Our reality has been irrevocably changed by a global COVID-19 pandemic that has been ongoing since the beginning of 2020. In the recent scientific literature, we find reports indicating that critically ill COVID-19 patients have increased risk of serious fungal infections, such as invasive pulmonary aspergillosis, invasive candidiasis, or *Pneumocystis jirovecii* pneumonia [3,4]. The latest news indicates that COVID-19 may also predispose to mucormycosis, a rare angioinvasive disease with high morbidity and mortality caused by multi-drug resistant fungi of the order *Mucorales* [5,6,7,8,9,10,11]. Prophylaxis of airborne infections includes the use of protective masks. In the era of a COVID-19 pandemic, masks have become mandatory almost all over the world, especially indoors. Wearing masks that are not always handled according to the manufacturer’s instructions and also frequent disinfection of the skin make us more susceptible to the development of diseases caused by various pathogens, including fungi. This applies in particular to the skin which, under the influence of alcohol-based disinfectants, loses part of its natural insulating components and natural skin microbiota that protects against the attack of pathogenic microorganisms. Similarly, moist and not often enough changed masks covering the nose and mouth are a perfect place for fungi to grow and may lead to the development of superficial or even respiratory mycoses. These facts indicate that among the social and economic problems that will remain for humanity to face after the pandemic, there may also be another epidemic—this time, one that is associated with fungal diseases.

The therapeutic options for fungal infections are very limited. The currently available antifungal drugs belong to only a few classes based on their mode of action—namely, polyenes that bind to ergosterol in the fungal cell membrane, leading to cell lysis, azoles, allylamines, and morpholine analog (amorolphine) that target ergosterol biosynthesis; echinocandins, which inhibit the synthesis of beta-glucan in fungal cell wall; analog of pyrimidine (flucytosine) that interferes with pyrimidine metabolism, RNA/DNA, and protein synthesis; and mitotic inhibitor (griseofulvine) that interferes with microtubule function. Many drugs have very limited use due to the route of administration, toxicity and side effects, and drug interactions, as well as due to bioavailability at the target site (e.g., penetration into brain tissue) [12]. As for treatment of most the common fungal infection, candidiasis, there have been several breakthroughs in recent years using prediction models for patients susceptible to such infections due to associated diseases or other immune system impairments [13,14].

The treatments associated with fungi are undoubtedly a complex issue; nevertheless, there is an undeniable need for new substances with antifungal activity. The development of new antimycotics that are cost-effective and active against a variety of fungi is not only an essential medical need but also a socio-economic necessity. In particular, access to established antifungal drugs is often limited to developed countries, while geographic and sanitary conditions make them most relevant to countries with lower incomes. The production of such drugs should be technically simple and not burdened with profit-driven restrictions imposed by big pharma, which in practice means the need to create a completely new concept. The concept of polymeric drugs fits into all this in a very good way.

Beta-glucans—a subgroup of polysaccharides that are distinguished by the type of bond connecting glucose molecules in the main polymer chain—have recently attracted particular interest in medicine [15]. There are two main postulated applications of beta-glucans related to the treatment of diabetes [16] and to use as immunomodulators [17]. It is speculated that beta-glucans may be one of the factors responsible for the lower incidence of cancer on the Asian continent, due to their presence in mushrooms, an important component of the Asian diet [18]. Beta-glucans, especially the ones that are not soluble in water, are mainly obtained from mushrooms [19]. Limited solubility in water may in the future become a problem in terms of a treatment’s route of administration, so it is reasonable to study the activity of charged beta-glucan derivatives whose solubility would be much better, increasing the chance that the biological activity will be retained. Particularly interesting here may be cationization, i.e., adding a positive charge to the structure of polymers using GTMAC. This is a frequently reported method in the literature to cationize polysaccharides for medical purposes [20]. The polysaccharides that result from this reaction often have antibacterial properties [21]. Combining these two facts, i.e., positive charge and origin from fungi, and thus resulting in a spatial structure similar to the natural component of the fungal cell wall, we can assume that cationic beta-glucans will have antifungal properties.

Polymers with a positive charge that can easily interact with a negatively charged biological membrane seem to be particularly promising in this regard [22]. This feature leads polycations to exhibit biocidal activity, especially bactericidal and bacteriostatic activity, which has been documented in literature [23]. In view of this observation, it seems reasonable to hypothesize here that this property will also apply to fungi. From the point of view of medical applications, more important than showing the antimycotic property itself is to demonstrate a polycation structure that will not be toxic to mammalian cells at concentrations that are toxic to fungal cells. This is exactly what we were able to show in this work involving obtained cationic derivatives of beta-glucans of *Saccharomyces boulardii* and *Cetraria islandica*.

## 2. Results

### 2.1. Chemical Characteristics of the Obtained Materials

#### 2.1.1. Elemental Analysis

The mass percentage of a given chemical element in the material in subsequent purification stages allows for a preliminary assessment of the effectiveness of the polysaccharides isolation from biological material. In the case of polysaccharides isolation procedures, the most undesirable group of contaminants is proteins. Elemental analysis allows determination of the amount of protein indirectly as the amount of nitrogen in the sample.

Comparing the preliminary and final stages of purification (data in Table 1) in the case of both sources, it can be concluded that the material was effectively deproteinized because the amount of nitrogen decreased. In the case of *C. islandica* (CI), where the amount of protein in the starting material was low from the beginning, it dropped to zero. In the case of *S. boulardii* (SB), containing more proteins, a negligible amount of nitrogen remained in the final material used for cationization, which may indicate a marginal quantity of proteins remaining in the sample. Cationization of polysaccharides is carried out by attaching a quaternary amine, which will cause an increase in the amount of nitrogen in the structure of the molecule. We observed this phenomenon also for our results, which confirmed the effectiveness of cationization.

#### 2.1.2. Zeta Potential of the Systems Studied

The zeta potential value is the most compelling evidence for the effectiveness of cationization. In the case of the new polymer structures proposed in this work, as we postulated, the positive charge of the molecule is a necessary condition for obtaining biological activity. Zeta potential measurements were carried out for all obtained polycations, which are a good approximation of the charge in the case of polyelectrolytes [Table 2]. Additionally, the zeta potential of the water-soluble substrate for the synthesis of the yeast-derived polysaccharide was measured to evaluate the charge of these polymers [Table 2].

#### 2.1.3. GPC Chromatography Assessment of Molecular Weight and Its Distribution

Polysaccharides occur in nature as a collections of macromolecules of different masses; therefore, when examining these polymers, one should always think in terms of a heterogeneous system of greater or lesser molecular weight dispersion. In this work we isolated mixtures of macromolecules and then obtained cationic derivatives of their systems, which is why assessment of mass and mass distribution using GPC chromatography is necessary. Full chromatograms are included in the Supplementary Materials (Figure S3) and a summary of the results is presented in Table 3.

According to the literature, the molecular weight of beta-glucans may range from 0.1 to 1000 kDa [24]; therefore, it can be said that the materials we received were in the middle of this range. Regardless of the source of raw substrate and method of cationization, we obtained materials with a similar mass, which may result from the fact that the cationization and isolation were carried out under similar moderate basic conditions in all cases (alkaline treatment was necessary due to the negligible solubility of most beta-glucans in water). This suggests that we were dealing with partially alkaline transformed beta-glucans. The second important parameter studied here was mass dispersion. In the case of the unmodified yeast beta-glucan, the results show that this simple isolation procedure gave a material that was surprisingly homogeneous in terms of weight distribution. The cationization of this compound worsened these parameters to some extent, which is to be expected in any reaction involving macromolecules not completely modified (not all OH groups present in the polymer react due to steric hindrance). In the case of SBBGTMAC2 and CIGTMAC, the mass dispersion was closer to that which would be expected for systems based on additionally modified natural polymers.

#### 2.1.4. Material Analysis Based on FT-IR Spectra

GPC gave us information about the molar masses of chemical compounds present in the tested samples, while more detailed data about the chemical composition (presence of functional groups) can be obtained from IR spectroscopy. We were dealing here with mixtures of similar compounds, so it was not possible to indicate directly complete structures, but it was possible to indicate with a high probability the chemical composition of the main components and potential impurities. In this case, these data allowed mainly for the assessment of material deproteinization at subsequent stages of purification (disappearance of bands at about 2850 cm^−1^ from NH_3_ ^+^ stretching oscillations, appearance of a broad well-defined bell-shaped band at about 3300 cm^−1^ from OH groups bound in hydrogen bonding) and confirmation of the successful cationization of polysaccharides (vibration of methyl groups in a quaternary amine at 1480 cm^−1^; no clearly separated peak, but an increase in absorbance on the slope of the stronger band is seen for this length). The graphs of the full FT IR spectra can be found in the Supplementary Materials (Figure S1 for SBB derivatives and Figure S2 for CI derivative).

### 2.2. Initial Assessment of Biological Properties of New Materials

#### 2.2.1. Antifungal Properties of the Obtained Polycations

The cationic beta-glucan derivatives were inactive against *Candida* strains. In the tested concentration range, polycations did not inhibit or weaken the growth of yeasts, and MIC values could not be identified. For filamentous fungi, the antifungal activity of the polymers was varied. The best antifungal effect was observed for *S. brevicaulis*, for which SBBGTMAC2 MIC was 62.5 mg/L, and SBBGTMAC MIC ranges were 250–1000 mg/L. For CIGTMAC, we noted a marked reduction in growth of the fungus relative to the growth control well without polycation at concentrations ≥ 3.9 mg/L, but complete growth inhibition did not occur. Similar results with significant reduction in fungal growth were obtained for SBBGTMAC2 against *A. brasiliensis* and *F. solani* for concentrations ≥ 125 mg/L and 250 mg/L, respectively. SBBGTMAC showed antifungal activity against two of the three tested *Aspergillus* species, i.e., *A. brasiliensis*, for which MIC values ranged 62.5–125 mg/L, and to a lesser extent *A. flavus*, the growth of which was diminished at concentrations ≥ 125–500 mg/L. The detailed results of the study of the antifungal properties of the polycations are presented in Table 4.

#### 2.2.2. Evaluation of the Toxicity of the Obtained Polycations In Vitro Using Fibroblast Cell Line

**Figure 1:**
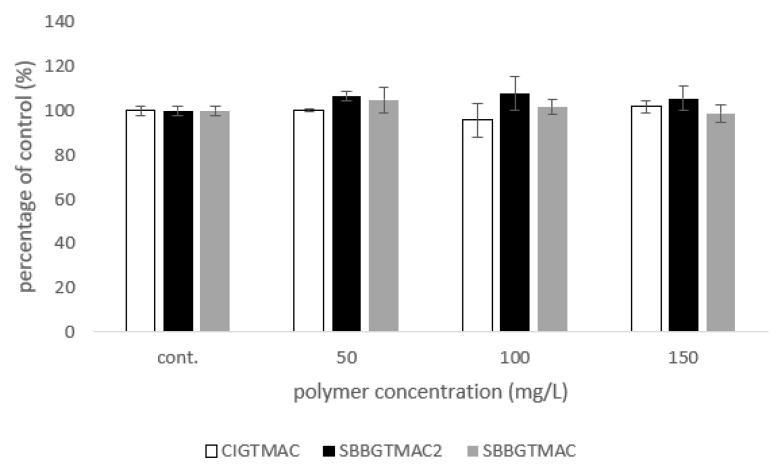
Preliminary toxicity studies of the obtained polycations using the 3T3-L1 fibroblast cell line. In the experiment, serum-free DMEM medium was used; exposure to the polymer lasted for 24 h.

A chemical compound that is a good candidate for an active ingredient in antimycotic formulations cannot exhibit universal biocidal properties. The toxicity towards eukaryotic cells, which should be low for such compounds, is especially important because it will let us assess, in advance, the potential side effects. Hence, an evaluation of the cytotoxicity of the obtained polycations against fibroblasts, the most common cells present in the mammalian body, was carried out (Figure 1).

The obtained data show that for concentrations up to 150 mg/L, we did not observe a negative effect on fibroblasts for all tested polymers (no statistically significant difference vs. control was found). We also did not observe any differences between the actions of individual cationic polysaccharides. Due to the large unavoidable differences in the culture conditions of the mammalian and fungal cells, the therapeutic index in the case of antifungal use of the polymers cannot be directly calculated here. However, using data from this and earlier subsections, it can be concluded that for concentrations safe for eukaryotic cells, we are observing a negative effect on the growth of selected strains of fungi for selected polymers (pairs *A. brasiliensis*/SBBGTMAC and SBBGTMAC2 or *S. brevicaulis*/SBBGTMAC2).

### 2.3. An In Vivo Model of Fungal Infection

For evaluation of the antifungal activity of the tested polymers using an in vivo infection model, only SBBGTMAC2 was chosen due to the best synthesis yield of this compound, accompanied by relatively high activity in preliminary studies (for two fungal strains). The insect model host *Galleria mellonella* combined with *A. brasiliensis* was used due to the fact that *G. mellonella* was described as a good model for testing *Aspergillus* pathogenicity and that in this case it can give us a reliable proof of concept [25,26,27]. These compromises in the scale of these experiments are due to the fact that these were preliminary studies, and the simplicity and efficiency aspect of the synthesis was assumed to be a priority in this work.

**Figure 2:**
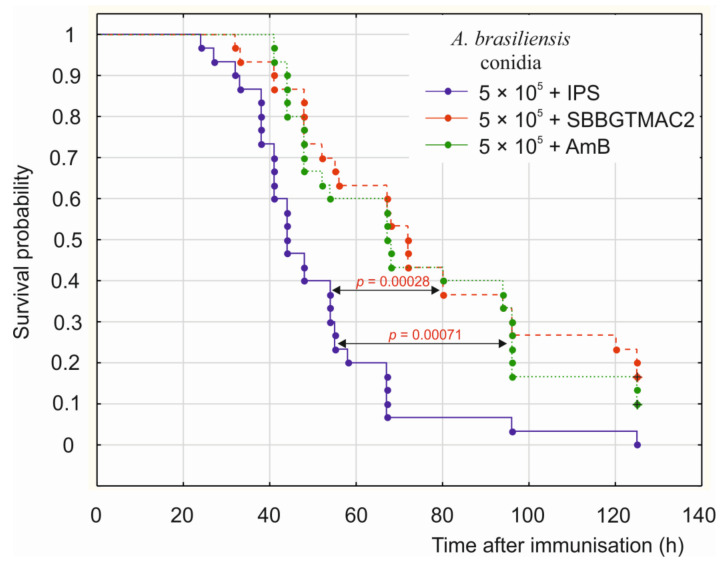
The effect of SBBGTMAC2 on survival of *G. mellonella* larvae infected with *A. brasiliensis*. The larvae were injected with suspension containing 5 × 10^5^ of *A. brasiliensis* conidia and, after 2 h-incubation, with IPS (control) or SBBGTMAC2 or AmB. The larvae survival was evaluated, and survival probability was estimated using the Kaplan–Meier estimator. Survivability comparisons between control and two other groups were done using log-rank test. The effects of SBBGTMAC2 and AmB were comparable, as no statistically significant difference was found between these groups. The graph shows results from three independent experiments.

The *G. mellonella* larvae were injected with live *A. brasiliensis* conidia at a dose of 5 × 10^5^ and then with SBBGTMAC2 at a concentration corresponding to an MIC value determined in vitro. The survival probability of the infected individuals treated with SBBGTMAC2 increased significantly in comparison with the control larvae treated with insect physiological saline (IPS) after infection with *A. brasiliensis* (*p* = 0.00028) (Figure 2). The survival analysis showed that the probability that larvae injected with *A. brasiliensis* and then IPS would survive 60 h was approximately 20%, whereas for the infected larvae treated with SBBGTMAC2 the probability was approximately 60%. Probability of survival of 120 h was approximately 5% and 25%, for the infected larvae treated with IPS and those treated with SBBGTMAC2, respectively. The effect of SBBGTMC2 on the insects’ survival was similar to that calculated for the amphotericin B (AmB)-treated larvae. All the larvae infected with *A. brasiliensis* and then injected with IPS were dead at the end of observation, i.e., at 125 h post-treatment. In contrast, at the same time, 17% and 10% of the larvae injected with SBBGTMAC2 and AmB, respectively, were still alive (Figure 2).

The survival experiments conducted using the insect model host *G. mellonella* indicated that the tested polycation SBBGTMAC2 exhibited in vivo antifungal activity against *A. brasiliensis* at a concentration corresponding to the MIC value determined in vitro. These results confirm the antifungal activity of SBBGTMAC2 in an in vivo model and, simultaneously, demonstrate the usefulness of this insect model for testing the antimicrobial activity of such polycationic molecules.

## 3. Discussion

Beta-glucans are polysaccharides found in the cell walls of certain bacteria, fungi, algae, lichens, and plants. They are used in medicine as natural preparations with antitumor properties, which decrease the levels of cholesterol and glucose in the serum and also stimulate the immune system [19,28]. The antibacterial properties of beta-glucans are also known and well described [19,20,21,22,23,24,25,26,27,28,29,30,31], but little is known about their direct action on fungi and possible direct antifungal activity. So far, research has focused on understanding the interactions between host cells (mainly animal) and beta-glucan and the host anti-fungal immunity induced by this compound [32].

This work describes the synthesis of three cationic beta-glucan derivatives using raw material isolated from *S. boulardii* and *C. islandica*. The purity of the materials obtained and the effectiveness of the cationization of the final products were confirmed. In the case of *S. boulardii*, two synthesis strategies were used, of which the one omitting the isolation of unmodified beta-glucan proved to be significantly more efficient (20 times higher yield SBBGTMAC2 vs. SBBGTMAC). In the case of these two derivatives, although the initial substrate for the synthesis was the same, we obtained polymers differing in their physicochemical properties—in particular, differences in their molecular weight and their dispersion (Table 3). These differences also resulted in noticeable differences in biological properties—in particular, antifungal properties expressed as MIC values (Table 4). However, these were not qualitative changes because, for the majority of tested strains, where one derivative has antifungal properties, the other also has them. Beta glucans are mixtures of macromolecules, and the method of isolation (or subsequent chemical modification) will affect the composition of the fractions that one obtains, and this is an inescapable problem with acquiring this type of natural compound.

These compounds have been investigated for their antifungal activity on selected fungal strains of yeasts and filamentous fungi. Beta-glucan derivatives showed selective in vitro antifungal activity against some molds—*S. brevicaulis*, *F. solani*, *A. brasiliensis*, and *A. flavus*—in the concentration range tested (1.95–1000 mg/L). The antimycotic properties of one of the cationic derivatives tested (SBBGTMAC2) were confirmed in *G. mellonella* larvae infected with *A. brasiliensis*. The administration of the compound resulted in a reduction in mortality, and the effect was similar to the result obtained for the known antifungal drug amphotericin B.

*Candida albicans* is still the most common etiological factor of candidiasis. Other Candida species cause infections at varying rates, often depending on the population (adults, children), but the rates also vary geographically. The choice of *Candida albicans* was obvious and due to epidemiological data. It was decided to test *Candida glabrata* and *Candida krusei* due to their natural resistance and reduced sensitivity to azole drugs commonly used in the prevention and treatment of candidiasis. The conducted studies were preliminary; therefore, the activity in relation to selected species of fungi was assessed. Other Candida species can be included in larger studies, but the obtained results do not indicate an antimycotic effect of tested beta-glucan derivatives on this genus. The applied methodology of antifungal activity testing of beta-glucan derivatives was not suitable for the assessment of the antimycotic effect on *Pneumocystis jirovecii*. This species does not grow in vitro in fungal culture media.

The results of our studies are promising and require research on more strains of different fungal species. Higher concentrations of beta-glucan derivatives should be investigated as well, which may prove to be fungistatic or fungicidal on more species. So far, mainly the antibacterial activity of beta-glucans has been examined. Studies of their various derivatives isolated from various organisms showed in vitro antibacterial activity at concentrations of 250 mg/L [29] or higher—1000–5000 mg/L [30]. In vivo studies also confirmed the protective effect of beta-glucan against various bacterial pathogens [33] but few clinical trials involving humans have been performed, and results remain controversial [34,35,36]. The studies conducted so far on glucan and fungi are sparse; however, they do indicate that this compound might have antimycotic activity, even against *Candida albicans*, which we did not show in our in vitro studies. Rice et al. demonstrated in an animal in vivo model that oral glucan administration in a dose of 1 mg/kg increased survival in mice challenged with *C. albicans* [37]. While Indian scientists found antifungal activity of β-D-glucan nanoparticles against *Pythium aphanidermatum*—a soil-borne plant pathogen [38]. It should be emphasized that the results of research on the antimicrobial activity of beta-glucans are difficult to compare with each other. This is due to the fact that these compounds are usually obtained from various organisms, subjected to various modifications, and therefore may show differences in biological activity. Our research is innovative in this field because it involves many different fungal species and also includes an in vivo model of fungal infection and beta-glucan-derivative activity. Obviously, far more studies are needed to confirm the antimycotic effects of cationic beta-glucan derivatives, but due to the growing need to search for new antifungals, such research seems even more significant and justified.

## 4. Materials and Methods

### 4.1. Chemistry

#### 4.1.1. General Instrumentation and Materials

*Saccharomyces boulardii* strain CNCM I-745 (Enterol 250, Biocodex, Poland), dried well-ground *Cetraria islandica* (vegetative aboveground part)—herbal raw material (ASKOM, Wroclaw, Poland), potato dextrose broth PDB (Sigma-Aldrich, St. Louis, MO, USA), glycidyltrimethylammonium chloride (technical, ≥90%, Sigma-Aldrich, St. Louis, MO, USA), sodium hydroxide p.a. (POCH, Gliwice, Poland), and hydrochloric acid, acetic acid (Idali, Radom, Poland) were obtained. All dialyses were performed using a cellulose tube (cut of 3.5 kDa) produced by Carl Roth Company (Karlsruhe, Germany). Poly(2-vinylpyridine) and PEG molecular weight standards PSS were obtained from PSS Polymer Standards Service GmbH, Mainz, Germany. Evaluation of elemental composition was commissioned on a Simultaneous CHNS combustion analyzer apparatus (Micro Cube elemental analyzer, Elementar Vario). Three independent measurements were performed for each sample; the result presented is the arithmetic mean ± standard deviation (SD) of the obtained results. IR spectra were measured using an FT-IR instrument (Nicolet iS10, SMART iTX, Thermo Scientific, Waltham, MA, USA).

#### 4.1.2. Culture Conditions for Saccharomyces Boulardii

The yeast was grown in potato dextrose broth medium (250 mg of inoculum lyophilisate per 1 L of medium) for 24 h at 25 °C with constant orbital shaking (200 rpm). An amount of 2 L of obtained yeast suspension was used for the isolation of beta-glucans.

#### 4.1.3. Isolation of Polymers from Biological Material

##### Saccharomyces Boulardii

Insulation of Raw Components of the Cell Wall

The isolations were carried out using a modified procedure described in a previously published work [39]. Briefly, 2 L of yeast suspension obtained from the culture was centrifuged at 500× *g* for 2 min. The supernatant was then gently removed, the remaining pellets were pooled in one vessel, and PBS buffer was added to the obtained suspension to obtain 10 mL. The suspension was cooled on ice and then, without removing it from the ice bath, it was subjected to an immersion source of ultrasound with a power of about 10 W (Ultrasonic Processor with a titanium sonotrode tip, Sonics, Vibracell). The lysis of yeast was performed with 8 repetitions consisting of a two-stage cycle: 5 min ultrasound, 2 min of mixing on ice to remove excess heat. The non-lysed yeast was removed by centrifuging the obtained suspension twice at 500× *g* for 2 min. The finally obtained supernatant was centrifuged at 1000× *g* at 4 °C for 20 min. The pellet composed of components of the cell wall was rinsed twice with sterile water, and then the vessel containing it was immersed in boiling water for 3 min to deactivate all present enzymes.

Extraction of Beta-Glucans

Material obtained as described in the previous subsection after cooling down to room temperature was suspended in 10 mL 2% sodium hydroxide, and then the mixture was heated to 90 °C for 5 h with constant stirring. After cooling, the suspension was centrifuged at 3000× *g* for 10 min, and the obtained supernatant was neutralized with 2 M acetic acid and mixed with 3 volumes of ethanol to precipitate the raw product. The pellets were rinsed with 2 mL of ethanol and dried under vacuum without heating. Then, the crude product was dissolved in 3% acetic acid and centrifuged (3000× *g* for 10 min) to remove the remaining proteins. The recovered supernatant was neutralized with 2 M NaOH and dialyzed against water (water change every 12 h to fresh and sterile) for 4 days. The finished product was obtained by freeze-drying the obtained solution. The yield of the resulting product was ~2%.

Material from *Cetraria islandica*

The isolation of beta-glucan from *C. islandica* was performed in a way similar to the procedures described previously [40,41]. Briefly, 10 g of dried well-ground *C. islandica* powder was suspended in 250 mL of ethanol (99%) with constant stirring for 1 h. The preliminary extraction of impurities was repeated twice. The brown-colored ethanol was then removed by filtration, and the solid was dried under reduced pressure without heating. Then the dry material was suspended in 100 mL of distilled water and heated to 100 °C. Extractions were carried out at this temperature for 4 h with continuous vigorous mixing. After this time, the still-warm mixture was centrifuged 3000× *g* for 10 min, and the resulting supernatant was poured into ethanol to precipitate the crude polymer (1 volume of supernatant to 2 volumes of ethanol). The resulting suspension was again centrifuged in an analogous manner to obtain a precipitate. The pellet was then dried under reduced pressure without raising the temperature. The last step of the purification consisted of redissolving the dry crude polysaccharide in water at 100 °C, hot centrifugation (3000× *g* for 10 min), and dialysis of the obtained supernatant against sterile distilled water. After 24 h of dialysis in the tube, polymer fibrous lumps had precipitated, which were filtered, rinsed with ethanol and dried at 40 °C under reduced pressure for 4 h. The yield of the resulting product was ~20%.

#### 4.1.4. Synthesis of Polycations

##### Using Purified Beta-Glucans

**Figure 3:**
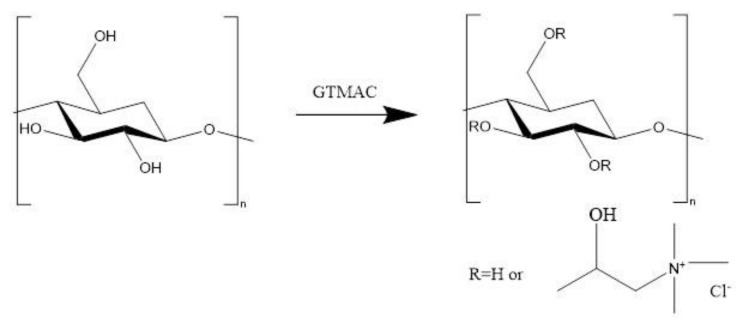
Scheme of beta-glucan cationization reaction used in this study.

For beta-glucans from both sources, the cationizations were performed in the manner used to obtain the cationic dextran described in our earlier work [20,42]. Briefly, 200 mg of beta-glucan was dissolved in 50 mL of distilled water containing an additional 200 mg of NaOH. The dissolution was carried out at 60 °C, and then 6 mL of GTMAC was added. The reaction was continued at this temperature for another 4 h, and then the mixture was cooled to room temperature and dialyzed against water. Final products were isolated from the obtained solutions by freeze-drying. The total yield of the resulting product was ~1% for SBBGTMAC and ~10% for CIGTMAC. A scheme of beta-glucan cationization reaction is presented in Figure 3.

##### Cationization of *Saccharomyces boulardii* Raw Cell Wall Components

Isolated as described in the previous section, raw components of the cell wall were suspended in 10 mL of sterile distilled water. After 1 day of dialysis (sterile water change every 12 h), the resulting suspension was centrifuged at 1000× *g* for 20 min. Then pellet was washed thoroughly with ethanol with two 10 mL portions and dried under reduced pressure without heating. Next, 200 mg of obtained material was dissolved in 50 mL of distilled water containing 200 mg of NaOH. Dissolution was carried out at 60 °C, and then 6 mL of GTMAC was added. The reaction was continued at this temperature for another 4 h, and then the mixture was cooled to room temperature and dialyzed against water. Obtained raw products were isolated from the obtained solutions by freeze-drying. Then this material was suspended in a mixture of 15 mL of ethanol and 5 mL of water and shaken for 4 h at room temperature. The resulting suspension was centrifuged at 1000× *g* at 4 °C for 20 min, and the product was isolated from the supernatant by evaporating the solvent using a rotary evaporator. The last step of purification consisted of dissolving the dry crude product in water and centrifugation at 1000× *g* for 20 min, and the final product was isolated from the supernatant by freeze-drying. The total yield of the resulting product SBBGTMAC2 was ~20%.

#### 4.1.5. Gel Permeation Chromatography (GPC) and Zeta Potential Measurements

The purpose of GPC measurements was to assess the average molecular weight and dispersion of the molecular weight for the obtained polymers [20,43] and thus to find out how heterogeneous was the beta-glucan mixture that was obtained [20,43]. Measurement was possible only in the case of the beta-glucans from yeast since the material in the obtained form dissolved in water. This polymer was composed of uncharged macromolecules (confirmed experimentally, details later in this chapter), and this is why the PEG standards were used to assess molecular mass (eluent composed of 0.1 M NaCl in water). In the case of the material derived from *C. islandica*, we obtained a polymer that was insoluble in water at a temperature below 80 °C, which made GPS measurements impossible in classical systems. In the case of products after cationization, they were all well soluble in aqueous solutions, so GPC measurements were performed. Due to the positive charge of these polymers (confirmed by measurements of the zeta potential), poly(2-vinylpyridine) standards were used for molecular mass determination (eluent consisting of 0.3 M Na_2_SO_4_ aqueous solution containing 0.5 M acetic acid). In all cases, the column PolySep™-SEC GFC-P Linear, LC Column 300 × 7.8 mm (Phenomenex, Torrance, CA, USA) was used; the flow rate, injection volume, and polymer concentration were, respectively, 0.8 mL/min, 100 µL, and 5 g/L. The measurement of the zeta potential was performed using a Zetasizer Nano ZS (Malvern Panalytical, Malvern, UK) apparatus and polymer solutions in demineralized water (pH 6.0, conductivity 0.5 µS) with a concentration of 5 g/L [20,44]. Three independent measurements were made for each sample; the result presented is the arithmetic mean ± standard deviation (SD) of the obtained results.

### 4.2. Preliminary Assessment of Cytotoxicity on Mammalian Cells

Embryo mouse fibroblast 3T3-L1 (ATCC CL-173) (Manassas, USA) was used to assess the toxicity of polycations. The medium for this cell line was Dulbecco’s modified Eagle’s medium (DMEM, Sigma-Aldrich, St. Louis, MO, USA), supplemented with fetal bovine serum (Sigma-Aldrich, St. Louis, MO, USA), for a final concentration of 10% (*v*/*v*) and 1% (*v*/*v*) penicillin–streptomycin solution (Sigma-Aldrich, St. Louis, MO, USA). Cultures were incubated at 37 °C in atmosphere containing 5% of carbon dioxide (CO_2_). Cultures were seeded 6 × 10^4^ cells per well in 24-well plates and grown for 24 h. After that, the medium was changed to serum-free, and cells were treated with polycations solution (in serum-free media) for the next 24 h to assess cytotoxicity using neutral red uptake assay [45]. Three independent measurements were made for each concentration; the result presented is the arithmetic mean ± standard deviation (SD)of the obtained results. Additionally, the lack of statistical differences in cell viability was confirmed using the Mann–Whitney U test.

### 4.3. Assessment of Antifungal Properties

#### 4.3.1. General Instrumentation and Materials

Strains for antifungal activity testing were *Candida albicans* ATCC 90028, *C. glabrata*, ATCC 15454, *C. krusei* ATCC 6258, *Aspergillus flavus* ATCC 204304, *A. brasiliensis* ATCC 16404, *A. fumigatus* clinical strain (Department of Infections Control and Mycology, Jagiellonian University Medical College culture collection), *Trichophyton mentagrophytes* ATCC 18748, *Fusarium solani* (Department of Infections Control and Mycology, Jagiellonian University Medical College culture collection), *Scopulariopsis brevicaulis* (Department of Infections Control and Mycology, Jagiellonian University Medical College culture collection). *Aspergillus fumigatus* and *Scopulariopsis brevicaulis* were identified using phenotypic methods based on fungal morphology [46]. *Fusarium solani* identification was established with matrix-assisted laser desorption ionization time-of-flight mass spectrometry (MALDI-TOF MS) [47]. Phenotypic identification was not confirmed by genetic methods.

We obtained RPMI-1640 medium with L-glutamine, without sodium bicarbonate (Sigma-Aldrich, St. Louis, USA); glucose (Chempur, Piekary Slaskie, Poland); 3-(N-morpholino) propanesulfonic acid (MOPS) (Glentham Life, Corsham, UK); flat-bottom polypropylene 96-well microdilution plates (VWR, Radnor, PA, USA); Sabouraud glucose agar with chloramphenicol: neopeptone (Difco Laboratories Inc., Franklin Lakes, NJ, USA); glucose (Chempur, Piekary Slaskie, Poland); agar (Biocorp, Waszawa Poland); chloramphenicol (Farm-Impex, Gliwice, Poland); Czapek yeast extract agar: ZnSO_4_ × 7H_2_O (POCH, Gliwice, Poland), CuSO_4_ × 7H_2_O (ACROS, Belgium); MgSO_4_ × 7H_2_O (POCH, Gliwice, Poland); KCl (Chempur, Piekary Slaskie, Poland); NaNO_3_ (POCH, Poland); saccharose (Chempur, Piekary Slaskie, Poland); K_2_HPO_4_ (Chempur, Piekary Slaskie, Poland); agar (Biocorp, Warszawa, Poland); and yeast extract (Oxoid, Pratteln, Switzerland).

Apparatuses included a densitometer (Biosan, Poland), incubator (POL-EKO, Poland), and vortex mixer (Labnet, Poland).

#### 4.3.2. Antifungal Activity Testing

The assessment of antimycotic activity of beta-glucan derivatives was performed using our own microdilution method in a liquid culture medium based on European Committee on Antimicrobial Susceptibility Testing methodology for antifungal susceptibility testing for yeasts and molds (EUCAST DEFINITIVE DOCUMENT E.DEF: 7.3.2. Method for the determination of broth dilution minimum inhibitory concentrations of antifungal agents for yeasts; 9.3.2. Method for the determination of broth dilution minimum inhibitory concentrations of antifungal agents for conidia forming moulds) and the Hancock Lab [48] procedure for cationic antimicrobial peptides.

##### Preparation of Stock Solutions and Working Solutions

Stock solutions were prepared in sterile distilled water to obtain concentrations of 10 g/L. The working solutions were prepared as a 2-fold dilution series of stock solutions in sterile distilled water.

##### Preparation of Microdilution Plates

Wells 1 to 10 of each column of the flat-bottom polypropylene 96-well microdilution plates were filled with 20 µL of the corresponding concentration of CIGTMAC, SBBGTMAC, and SBBGTMAC2, while each well of columns 11 and 12 was filled with 20 µL of sterile distilled water.

##### Inocula Preparation

The inocula were prepared from fresh cultures (24 h for yeasts, 2- to 7-day-old for filamentous fungi) on solid agar media—Sabouraud glucose agar with chloramphenicol for *Candida* species and *Trichopyton mentagropytes* and Czapek yeast extract agar for molds. Yeasts inocula were prepared by suspending a few representative colonies in sterile distilled water. Filamentous fungi colonies were covered with approximately 5 mL of sterile water, then the conidia were rubbed with a sterile cotton swab and transferred to a sterile tube. The suspensions were homogenized with a gyratory vortex mixer, and the cell density was adjusted to 0.5 McFarland. The inocula were used for testing within 30 min of preparation.

##### Preparation of Working Suspensions

The working suspensions were prepared by dilution of the primary inocula in RPMI-1640 medium with L-glutamine, without sodium bicarbonate, with 2% glucose, buffered to pH 7 with MOPS (0.165 mol/L). Next, 20-fold dilutions were prepared, which gave the following inocula densities: 0.5–2.5 × 10^5^ CFU/mL for yeasts and 1–2.5 × 10^5^ CFU/mL for filamentous fungi.

##### Inoculation of Microdilution Plates

The microdilution plates were inoculated with 180 µL of the working suspensions, except sterility control wells, which contained 180 µL of microbial-free RPMI-1640 with L-glutamine, without sodium bicarbonate, with 2% glucose, buffered to pH 7 with MOPS (0.165 mol/L). The range of final concentrations of beta-glucan derivatives on microplates after the addition of the fungal suspension was 1.95–1000 mg/L.

##### Incubation of Microdilution Plates

The microdilution plates were incubated without agitation at 37 °C (yeasts) or 27 °C (molds, dermatophyte) in ambient air for 24–48 h.

##### Reading Results

The antimicrobial activity was estimated visually. Minimal inhibitory concentration (MIC) values of tested compounds were defined as no visible growth of fungi by eye.

### 4.4. Galleria Mellonella Larvae Survival Experiments

The last instar larvae (250–300 mg weight) of the greater wax moth *Galleria mellonella* (Lepidoptera: Pyralidae) from a continuous laboratory culture were used. They were maintained at 30 °C in the dark and reared on honeybee nest debris. The larvae were injected with 3 µL of insect physiological saline (IPS; 0.1 M Tris–HCl pH 6.9, 150 mM NaCl, 5 mM KCl) containing *Aspergillus brasiliensis* conidia (5 × 10^5^ per larva). After 2 h incubation at 37 °C, the larvae were randomly divided into three groups (10 larvae per group) and injected with 3 µL of IPS (control) or 3 µL of SBBGTMAC2 solution in IPS (80 μg per larva—the final concentration in larval hemolymph corresponded to an MIC value 1000 mg/L) or 3 µL of AmB solution (2 μg per larva in 30% DMSO; final concentration of DMSO in larval hemolymph—approximately 1.25%). The larvae were incubated at 37 °C, and survival was checked until 125 h after treatment. The entire experimental layout was repeated at three independent occasions (in total 90 larvae were used). The larvae survival was evaluated, and survival probability was estimated using the Kaplan–Meier estimator [49,50]. Survivability comparisons between control and the two other groups were done using the log-rank test. The graph shows results from three independent experiments.

## 5. Conclusions

Fungi and fungal-related diseases are a significant medical and socio-economic problem. The possibilities of combating fungi are currently very limited, and the search for new, effective, and inexpensive methods of their elimination is a very current need. Fungi are a heterogeneous group of organisms, which makes it difficult to find a compound showing a broad antifungal spectrum that in addition has low toxicity to humans, animals, and plants. Our research on antifungal activity of cationic beta-glucan derivatives indicates that these substances exhibit selective antifungal activity against certain species. No antifungal activity was found for unicellular yeasts of the genus *Candida*, while antimycotic activity against some filamentous fungi, i.e., *Scopulariopsis brevicaulis*, *Aspergillus brasiliensis*, and *Fusarium solani*, was shown. *S. brevicaulis* and *F. solani* are multi-resistant species, showing high MIC values for most of the antifungals currently available. The obtained promising preliminary results for these fungi encourage research to be conducted on a greater number of strains of these species and to extend the research to other species of filamentous fungi resistant to currently used drugs. Our research indicates that these compounds may not necessarily completely inhibit the growth of fungi, but the compounds can significantly limit it, so they could be successfully used as agents preventing the growth of fungi. In addition, the known immune-stimulating effect may potentiate the antifungal activity.

## Figures and Tables

**Table 1:** Elemental composition in the obtained materials at subsequent stages of isolation and modification of polysaccharides. The results are presented as mean of three independent measurements ± standard deviation (SD).

	The Elemental Composition of the Obtained Material (%)			
	N	C	H	S
Freeze-dried *Saccharomyces boulardii* (SB)	6.02 ± 0.03	44.44 ± 0.03	6.80 ± 0.04	0.00 ± 0.01
Dry lysed SB pellet (lSBp)	5.33 ± 0.04	42.18 ± 0.18	6.75 ± 0.01	0.00 ± 0.01
Purified beta-glucan from SB (SBB)	0.32 ± 0.01	38.60 ± 0,01	6.31 ± 0.10	0.00 ± 0.01
SBB cationized using GTMAC (SBBGTMAC)	1.44 ± 0.03	41.65 ± 0.05	7.04 ± 0.02	0.00 ± 0.01
SB raw cell wall components cationized using GTMAC (SBBGTMAC2)	6.68 ± 0.17	45.76 ± 0.24	7.60 ± 0.01	0.00 ± 0.01
Dried and well ground *Cetraria islandica* (CI)	0.22 ± 0.01	40.55 ± 0.01	6.58 ± 0.06	0.70 ± 0.02
CI after ethanol impurities extraction (EPCI)	1.45 ± 0.02	43.81 ± 0.23	6.45 ± 0.09	0.00 ± 0.01
Product of initial CI warm water extraction (IECI)	0.03 ± 0.01	36.21 ± 0.13	6.27 ± 0.12	0.00 ± 0.01
Purified beta-glucan from CI (BCI)	0.00 ± 0.01	36.16 ± 0.23	6.75 ± 0.14	0.00 ± 0.01
CI cationized using GTMAC				
(CIGTMAC)	2.35 ± 0.02	41.92 ± 0.11	7.53 ± 0.01	0.00 ± 0.01

**Table 2:** Zeta potential values measured for crude polysaccharide and corresponding cationic derivatives. The results are presented as mean of three independent measurements ± standard deviation (SD).

	SBB	SBBGTMAC	SBBGTMAC2	CIGTMAC
Zeta potential (mV)	−5.31 ± 0.56	32.93 ± 0.35	43.77 ± 1.05	36.16 ± 0.40

**Table 3:** The molecular weight determined from GPC measurements for crude polysaccharide and corresponding cationic derivatives.

	SBB	SBBGTMAC	SBBGTMAC2	CIGTMAC
Average molecular weight (kDa) (based on GPC)	24.95 *	29.30 **	23.92 **	53.48 **
Mass dispersion index	1.1	1.2	1.5	1.5

* PEG as mass standard; ** PVP as mass standard.

**Table 4:** MIC values (mg/L) for CIGTMAC, SBBGTMAC2, and SBBGTMAC against various fungal species. The results obtained from three replications are presented.

Name of Fungal Strain		MIC (mg/L)	
	CIGTMAC	SBBGTMAC2	SBBGTMAC
*Candida albicans* ATCC 90028	>1000	>1000	>1000
*Candida glabrata* ATCC 15454	>1000	>1000	>1000
*Candida krusei* ATCC 6258	>1000	>1000	>1000
*Aspergillus flavus* ATCC 204304	>1000	>1000	>1000 concentration ≥ 125–500 mg/L—diminished growth compared with control
*Aspergillus fumigatus*	>1000	>1000	>1000
*Aspergillus brasiliensis* ATCC 16404	>1000	>1000 concentration ≥ 125 mg/L—diminished growth compared with control	62.5–125
*Trichophyton mentagrophytes* ATCC 18748	>1000	>1000	>1000
*Fusarium solani*	>1000	>1000 concentration ≥ 250 mg/L—diminished growth compared with control	>1000
*Scopulariopsis brevicaulis*	>1000 concentration ≥ 3.9 mg/L—diminished growth compared with control	62.5	250–1000

## Data Availability

Not applicable.

## Supplementary Materials

The following are available online at www.mdpi.com/article/10.3390/ph14090838/s1, Figure S1: IR spectra of various stages of SBB purification (left) and products of beta-glucan modification using GTMAC (right), Figure S2: IR spectra of various stages of CI purification (left) and products of beta-glucan modification using GTMAC (right), Figure S3: GPC/SEC chromatogram of a beta-glucan isolated from *Saccharomyces boulardii* (left) and the obtained cationic beta-glucans (right).
