# Supplementary material for: Synthesis and Study of Antifungal Properties of New Cationic Beta-Glucan Derivatives
Source: Pharmaceuticals (Basel). 2021 Aug 24;14(9):838. doi: 10.3390/ph14090838 (PMC8469811; doi:10.3390/ph14090838)
Supplement: Supplementary file 1 [file pharmaceuticals-14-00838-s001.zip › pharmaceuticals-1327902-supplementary.pdf]

Supplementary Materials

Synthesis and study of antifungal properties of new cationic beta-glucan derivatives

Kamil Kaminski [1,*], Magdalena Skora [2], Paweł Krzyściak [2], Sylwia Stączek [3], Agnieszka Zdybicka-Barabas [3], Małgorzata Cytryńska [3]

[1] Jagiellonian University, Faculty of Chemistry, Gronostajowa 2 St., 30-387 Krakow, Poland;
kaminski@chemia.uj.edu.pl
[2] Jagiellonian University Medical College, Chair of Microbiology, Department of Infections Control and Mycology, Czysta 18 St., 31-121 Krakow, Poland; magdalena.skora@uj.edu.pl
[3] Department of Immunobiology, Institute of Biological Sciences, Faculty of Biology and Biotechnology, Maria Curie-Sklodowska University, Akademicka 19 St., 20-033 Lublin, Poland; cytryna@poczta.umcs.lublin.pl
* Correspondence: kaminski@chemia.uj.edu.pl

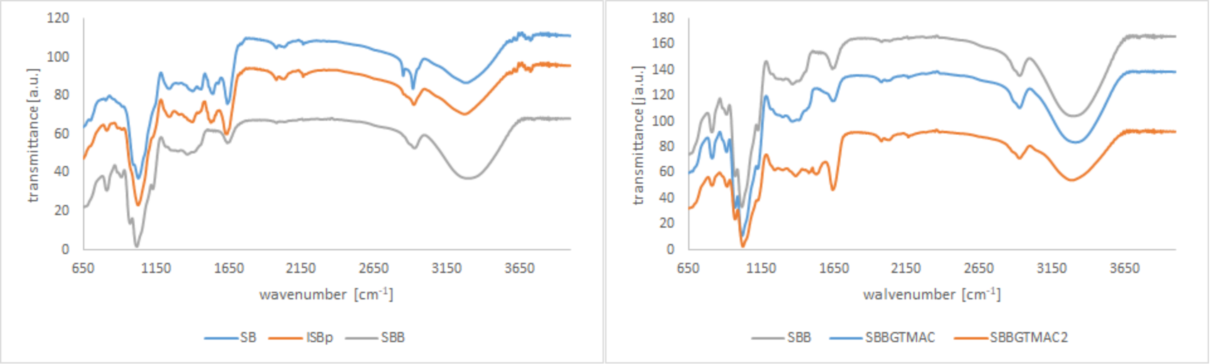

Figure S1. IR spectra of various stages of SBB purification (left) and products of beta-glucan modification using GTMAC (right). Sample names : freeze-dried Saccharomyces boulardii (SB); dry lysed SB pellet (ISBp); purified beta-glucan from SB (SBB); SBB cationized using GTMAC (SBBGTMAC); SB raw cell wall components cationized using GTMAC (SBBGTMAC2)

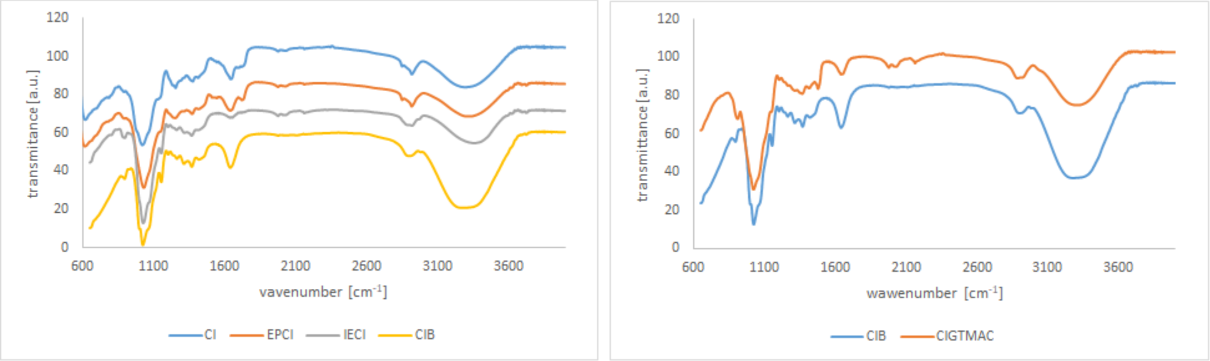

Figure S2. IR spectra of various stages of CI purification (left) and products of beta-glucan modification using GTMAC (right). Sample names : dried and well ground Cetraria islandica (CI); CI after ethanol impurities extraction (EPCI); product of initial CI warm water extraction (IECI); purified beta-glucan from CI (BCI); CI cationized using GTMAC (CIGTMAC).

In case of IR spectra obtained data allow mainly for assessment of biological material deproteinization at subsequent stages of purification and confirmation of effective cationization. In the case of SB (*Saccharomyces boulardii*) pretreatment by cell lysis only causes the 2850 cm^{-1} from $NH_3{}^{+}$ stretching oscillations bands (originating from proteins) to fade. In the case of the final purified beta glucan obtained from this material, we have the additional fading of bands at approximately 1550cm^{-1} (N-H) and 1400cm $^{-1}$(C-N) (also originating from proteins). All spectra in this case contain bands at 1650 cm^{-1} (C=O) present in all polysaccharides and proteins. In addition, deproteinization is evidenced by the presence, in the final purified material, of a broad well-defined bell-shaped band at about 3300 cm^{-1} from OH groups bound in hydrogen bonding.

Comparing purified beta glucans and the products of its cationic modification in case of SB the spectra differ mainly in the bands at 1480 cm^{-1} (vibration of methyl groups in a quaternary amine no clearly separated peak but an increase in absorbance on the slope of the stronger band is seen for this length) which confirms the effective cationic modification in conjunction with the elemental analysis data.

The changes in spectra during purification of CI (*Cetraria islandica*) are similar to materials obtained from SB but not as significant due to the smaller amount of protein in this crude material (based on elemental analysis). Greatest changes occur within the bands at 2850 cm^{-1} ($NH_3{}^{+}$stretching) and bell-shaped band at about 3300 cm^{-1}. Comparing purified beta glucans and the products of its cationic modification, the spectra differ mainly in the bands at 1480 cm^{-1} (vibration of methyl groups in a quaternary amine clearly separated peak compared to SB).

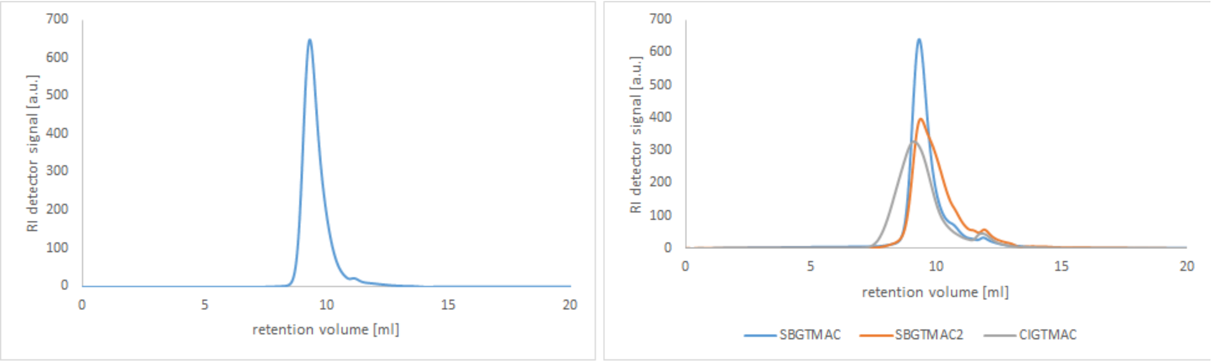

Figure S3. GPC/SEC chromatogram of a beta-glucan isolated from Saccharomyces boulardii (left) and the obtained cationic beta-glucans (right).
